# Au-Based Catalysts: Electrochemical Characterization for Structural Insights

**DOI:** 10.3390/molecules21030261

**Published:** 2016-02-25

**Authors:** Valentina Pifferi, Carine E. Chan-Thaw, Sebastiano Campisi, Anna Testolin, Alberto Villa, Luigi Falciola, Laura Prati

**Affiliations:** Dipartimento di Chimica, Università degli Studi di Milano, via C. Golgi 19, 20133 Milano, Italy; valentina.pifferi@unimi.it (V.P.); carine.chanthaw@unimi.it (C.E.C.-T.); sebastiano.campisi@unimi.it (S.C.); anna.testolin@studenti.unimi.it (A.T.); alberto.villa@unimi.it (A.V.); luigi.falciola@unimi.it (L.F.)

**Keywords:** bimetallic catalyst characterization, cyclovoltammetry, electrochemical impedance spectroscopy, AuPd catalysts, synergistic effect

## Abstract

Au-based catalysts are widely used in important processes because of their peculiar characteristics. The catalyst performance depends strongly on the nature and structure of the metal nanoparticles, especially in the case of bimetallic catalysts where synergistic effects between the two metals can be occasionally seen. In this paper, it is shown that electrochemical characterisation (cyclovoltammetry CV and electrochemical impedance spectroscopy EIS) of AuPd systems can be used to determine the presence of an electronic interaction between the two metals, thus providing a strong support in the determination of the nature of the synergy between Au and Pd in the liquid phase oxidation of alcohols. However, it seems likely that the strong difference in the catalytic behavior between the single metals and the bimetallic system is connected not only to the redox behaviour, but also to the energetic balance between the different elementary steps of the reaction.

## 1. Introduction

Gold nanoparticles have attracted more and more interest due to their special activity in catalytic reactions such as low-temperature oxidation of CO, hydrochlorination of alkyne, liquid phase oxidation of alcohols and polyols, *etc.* [1]. However, concerning selective liquid phase oxidation, despite the peculiar selectivity shown, gold catalysts suffer from a severe limitation consisting in the use of a basic environment. This drawback has a strong impact on the use of gold catalysts and promoted the evolution of monometallic into bimetallic systems. Indeed, Au in recent years has been used as a modifier of more active metals, even in homogeneous catalysis (Pd [2,3,4,5,6,7]; Pt [8,9]; Ru [10,11,12]; Ag [13,14]; Cu [15], *etc.*). In particular, the addition of Au to Pd or Pt catalysts in the liquid phase oxidation of polyols (sorbitol and glycerol) in the presence of O_2_ under mild conditions (<60 °C and <4 atm) not only improves catalytic activity and selectivity to the desired product, but also enhances the resistance to poisoning [16]. The presence of a synergistic effect has been connected to an intimate contact between the two metals and possibly to an isolation of the effective active sites [17,18]. However, the full understanding of Au-metal synergistic effects still remains uncertain because of two different reasons. The first one lies in the difficulties encountered in synthesizing bimetallic systems showing a homogeneous structure (alloy, core-shell, decorated nanoparticles) and composition throughout the whole sample. Indeed, segregation of metals and/or different ratio of metals present in the nanoparticles are the problems that often affect the preparation of these systems [19,20]. The second reason, even sometimes connected with the first one, is the lack of full characterization of a significative sample. HRTEM connected with EDX analyses and sometimes correlated with cross sectional analysis can be helpful in determining the structure and composition of nanoparticles [1,21,22], but are often performed on a few nanoparticles with the risk of not being representative of the whole sample.

The electrochemical behavior of the nanoparticles have been recently introduced as an important tool to determine the structure of nanoparticles [23,24] with the advantages of being practical and providing an overlook of a significative amount of sample. Therefore, we used Cyclic Voltammetry (CV) and Electrochemical Impedance Spectroscopy (EIS) techniques to characterize AuPd bimetallic nanoparticles and to establish a correlation between their electrochemical behavior and catalytic performances in the selective oxidation of glycerol. AuPd bimetallic nanoparticles prepared by coreduction allowed us to have alloyed samples which present an evident synergistic effect between Au and Pd, as already reported [25]. CV and EIS clearly pointed out that the electronic behavior of both metals are profoundly modified when alloyed, showing a lower potential of oxide reduction with respect to single Pd and higher than Au. This finding states that the electron transfer resistance is Pd > AuPd > Au. For explaining the higher catalytic activity of the AuPd alloyed particles compared to the ones of Pd and Au in catalytic oxidations, all the elemental steps have to be considered. In particular, according to experimental evidences [26,27] and kinetic models [28,29] that exclude a radical mechanism, the metal-catalyzed liquid phase oxidation of alcohols follows the dehydrogenation mechanism, which consists in a first step of hydride abstraction with formation of a metal hydride and in a second step of oxidation [30]. It is well-known that hydride species formation is favored in the case of Pd, while it constitutes a high energy demanding step in the case of Au [31]. We therefore concluded that in the AuPd catalyzed reaction the presence of alloyed nanoparticles allow a balance between the first step (Pd-favorite) and the second step (Au-favorite). Our findings are in agreement with the hypothesis of the presence of isolated Pd sites [32] with different catalytic properties with respect to bulk Pd and with high activity of Au in oxygen transfer reaction [33]. CV and EIS evaluations are moreover simple tools for evaluating the presence of an electronic interaction between the two metals.

## 2. Results

**Figure 1:**
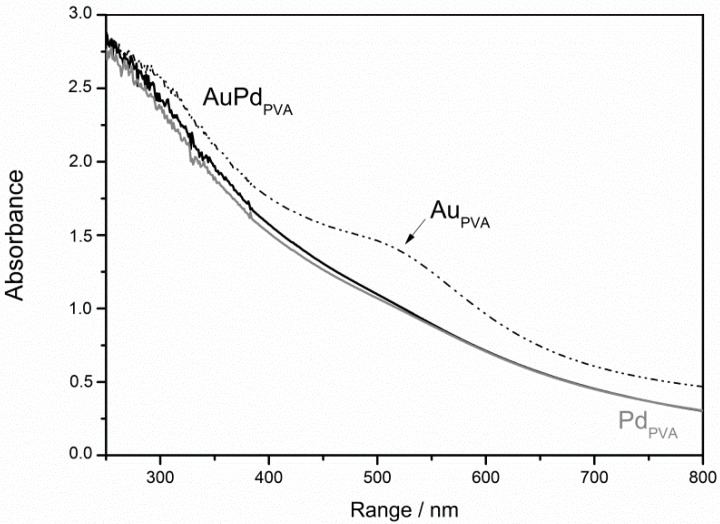
UV-vis spectra in H_2_O of Au_PVA_, Pd_PVA_ and AuPd_PVA_ nanoparticles.

Metallic sols of single Pd, Au and bimetallic AuPd were prepared accordingly to a well-known procedure [25] that provides a narrow size distribution within the 2.5–3.5 nm range (Table 1). In particular, the bimetallic samples deriving from coprecipitation/reduction of Au(III) and Pd(II) precursors have been confirmed to be fully reduced (UV-Vis spectrum, Figure 1) and did not show any Au segregation evident from the absence of the typical plasmonic resonance band of Au nanoparticle centered at 530 nm (Figure 1). TEM-EDX analyses confirmed the presence of the alloy.

**Figure 2:**
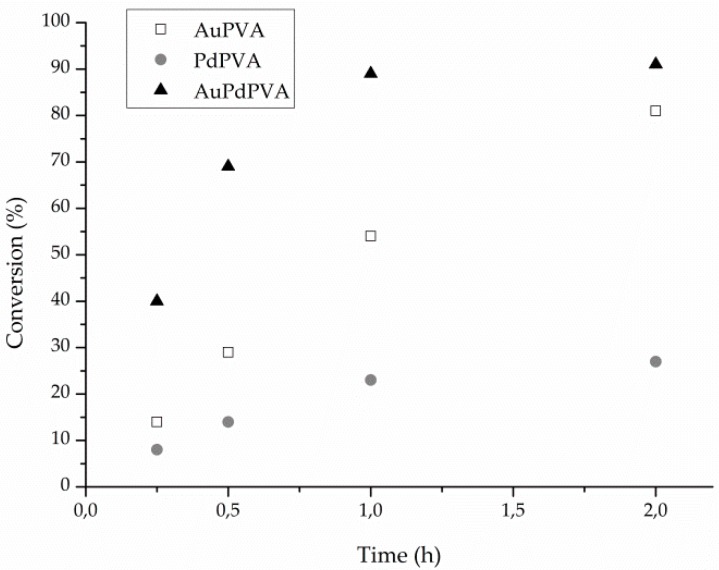
Reaction profiles for Au_PVA_, Pd_PVA_ and AuPd_PVA_ nanoparticles.

The monometallic and the bimetallic sols were tested in the liquid phase of glycerol. The results are shown in Table 1. As expected from the literature [34] the bimetallic nanoparticles showed the highest activity compared with the monometallic counterparts and a selectivity very close to that of Pd (Table 1). Moreover, the alloyed nanoparticles allow the conversion to proceed, reaching >90% conversion in only one hour (Figure 2). Conversely, monometallic Pd activity declined very rapidly with conversion stopping around 20%. Monometallic Au catalyst appears very stable (Figure 2). Moreover, AuPd system showed a selectivity more similar to Pd than to Au, the latter promoting the C–C bond cleavage and the subsequent higher selectivity to C2 (glycolic acid, 23%, and oxalic acid, 4%) and C1 (formic acid, 5%) products. The supposed reason for the extraordinary behavior of alloyed particle lies on the possible formation of Pd isolated sites, the chemistry of which differs from the one of Pd nanoparticles [32]. The detection of electronic effect on the single metals in the alloyed sample is complicated by the partial overlapping of Au and Pd XPS signals.

**Figure 3:**
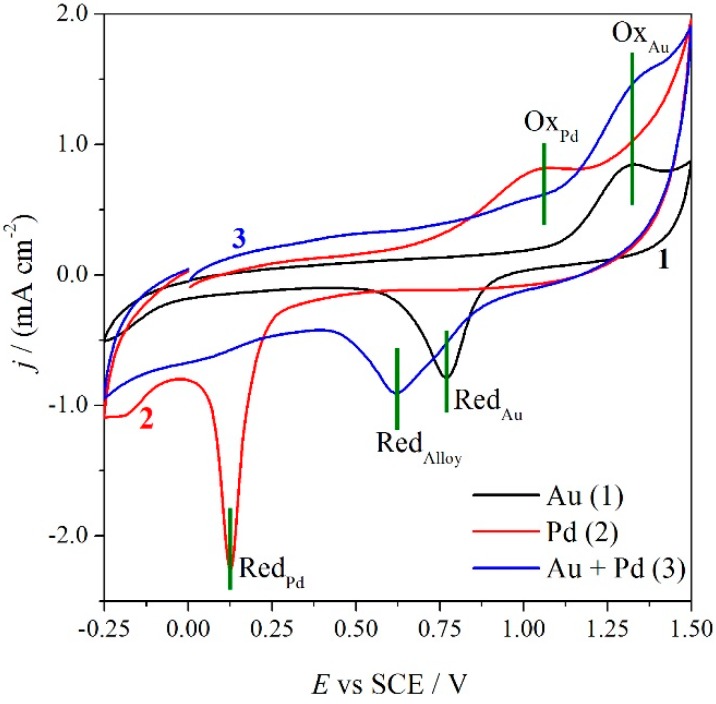
Cyclic Voltammetry of GC electrode modified with AuNP (1), PdNP (2) and Au-Pd alloy (3), registered in 0.1 M sulphuric acid at a sweep rate of 0.5 V·s^−1^.

We then characterized the three catalysts by CV and EIS in order to highlight any significative differences in redox properties. Figure 3 shows the voltammetric patterns, recorded in 0.1 M sulphuric acid at 0.5 V·s^−1^, of the AuPd nanoparticles casted on a glassy carbon inert electrode. The voltammetric features of palladium and the gold nanoparticles are also reported for comparison.

Gold and palladium nanoparticles present the characteristic oxidation (Ox_Pd_ and Ox_Au_) and reduction (Red_Au_ and Red_Pd_) peaks related to the formation and reduction of the surface metal oxides [24,35,36,37]. Palladium voltammogram is shifted in the cathodic region, indicating the higher stability of the palladium oxide with respect to the gold one. The voltammetry of the nanoalloy presents a larger oxidation peak due to the oxidation of the alloy, which comprises the two oxidation peaks of the single metals. On the other hand, the reduction voltammetric peak at 0.63 V, indicates that the oxide formed on the alloy is more stable with respect to the gold one, but really less stable in comparison with palladium oxide. In particular, it is worthwhile noticing that the nanoalloy metallic nature is restored at a lower energy with respect to palladium. It should be also noted that in the nanoalloy reduction scan, the presence of the two reduction peaks (Red_Au_ and Red_Pd_) related to partially segregated metals is still visible even if in a very low amount. This is in agreement with the observation made by Schiffrin for alloys containing a Pd percentage > 15% [24].

**Figure 4:**
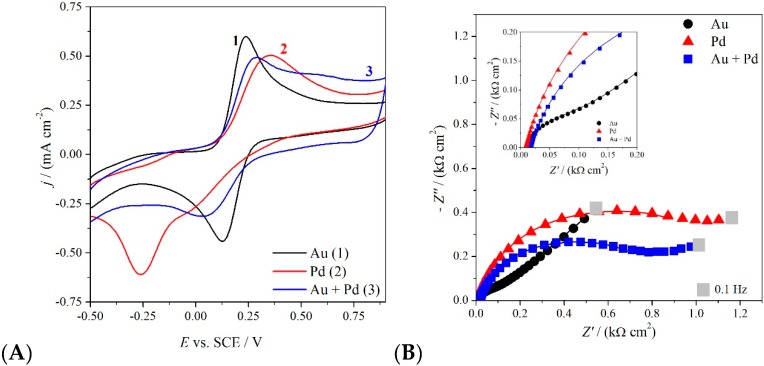
Cyclic voltammetry at 0.1 V·s^−1^ (**A**) and impedance Nyquist plots taken at 0.25 V (**B**) of GC electrode modified with AuNP (1), PdNP (2) and Au-Pd alloy (3), in the presence of 3 mM K_4_[Fe(CN)_6_] in 0.1 M KCl.

Figure 4 shows the voltammetric curves (4A) and the impedance Nyquist plots (4B, taken at 0.25 V) of the conventional Fe(CN)_6_^3−/2−^ redox probe registered in aqueous 0.1 M KCl at 0.1 V·s^−1^, for the three electrodes modified with Au, Pd and Au-Pd alloy.

Palladium nanoparticles present an electrochemical irreversible behavior, *i.e.*, very slow electrode kinetics with respect to the analyte mass transfer. On the other hand, gold nanoparticles show a faster electron transfer, as demonstrated by the electrochemical quasi-reversible CV feature (*E*_p_-*E*_p/2_ = 72 mV·s^−1^; Δ*E*_p_ = 128 mV·s^−1^). The Au-Pd alloy behaves intermediately, shifting the Pd irreversibility towards quasi-reversibility, thanks to the Au-Pd interaction. Analogues considerations can be driven considering the impedance spectra (Figure 4B), fitted with an equivalent circuit composed by the solution resistance in series with the charge transfer resistance and the double layer capacitance in parallel, in the case of Pd and the Au+Pd alloy, with the addition of a Warburg element in the Au case. The values of the charge transfer resistance *R*_CT_ (derived from the amplitude of the plot semicircles and accounting for the kinetics of the electronic transfer) are in the order Pd > Alloy >> Au, confirming the intermediate behavior shown in the voltammetric characterization.

## 3. Discussion

The electrochemical characterization of AuPd nanoparticles compared to that of single Pd or Au nanoparticles highlighted an intermediate behavior of the bimetallic nanoparticles both in terms of electron transfer resistance (CV and EIS) or in the stability of oxides (CV). The cyclic voltammetry and the impedance plots for AuPd are both able to clearly distinguish the presence of the alloyed species pointing out a net electronic interaction between the two metals. This electronic modification that involves both metals could be the base for the extraordinary synergetic effect detected during the use of the bimetallic systems as catalyst in the liquid phase oxidation of glycerol. In fact, Friend and coworkers [33] established that Au covered by oxygen atoms readily reacts with alcohols and accordingly, CV establishes that the oxide is not very stable compared to the Pd one. On the other hand, Pd oxide appears more stable than the AuPd one and this is in accordance with the study of Goodman and coworkers [32] that addressed the higher activity of AuPd with respect to Pd to a diluting effect of Au which is able to create catalytic single sites of Pd with a different reactivity.

Our study, however, pointed out that just on the basis of the relative stability of the metal oxides established by CV and the electronic transfer determined by CV and EIS it would be possible to conclude that Au is expected to be more active than Pd, but also than AuPd. Catalytic data showed that AuPd are by far more active than Pd, but also than Au.

Looking at the reaction profile (Figure 2) we can address the deactivation of Pd catalyst to a progressively covering of Pd surface by the oxide. In fact, CV shows that the PdO is highly stable (Figure 3). Conversely the Au reaction profile (Figure 2) did not show any deactivation and this behavior can be addressed to the poor stability of the oxide. AuPd reaction profile presented the highest activity together with a high stability (Figure 2) but an intermediate stability of oxide that is formed at more or less the same potential as the one on Au (Figure 3), but it is reduced at a lower potential than Au.

**Scheme 1:**
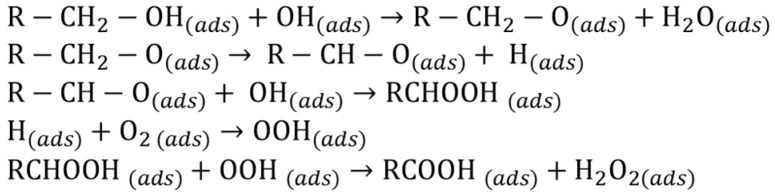
Alcohol oxidation mechanism.

Therefore, to explain the catalytic behavior of the AuPd system one should consider the whole catalytic cycle. The metal catalyzed oxidation mechanism follows a dehydrogenation path (Scheme 1) where the rate determining step for gold catalyst is the hydride abstraction from alcohol [26,27,28,29]. This step is more readily carried out by Pd, being the Pd-hydride species more stable than Au-hydride [38]:

The quite high stability of PdO which can be formed during the reaction can be invoked as the main cause of deactivation, normally observed with Pd catalyst. In the case of AuPd, the electronic modification to the Pd catalyst made by Au, decreases the stability of the oxide thus increasing the stability of the catalyst. On the other hand, the presence of Pd in AuPd bimetallic system allows the intermediate hydride to form, thus improving the overall reactivity.

Also from a selectivity point of view the AuPd system is more similar to Pd (Table 1), showing a lower selectivity towards C1 and C2 products (deriving from the C−C bond cleavage) compared to monometallic Au catalyst. This might be correlated to the enhanced accumulation of H_2_O_2_, responsible for C−C cleavage [39], in the case of Au, according to experimental observations [40] and to the higher calculated barrier for the peroxide decomposition on Au as compared to Pd. [41]. These considerations let us suppose that the most important effect to be considered is the modification that Au imparts to Pd.

## 4. Materials and Methods

### 4.1. Monometallic Nanoparticles

#### 4.1.1. Au nanoparticles Preparation.

Solid NaAuCl_4_•2H_2_O (Aldrich, Milan, Italy, 99.99% purity; 0.043 mmol) and polyvinylalcohol solution (PVA, MWPVA = 13,000–23,000, 87%–89% hydrolysed, Aldrich; 1% *w*/*w*; Au/PVA 1:1, *w*/*w*) were added to 100 mL of H_2_O. After 3 min, NaBH_4_ (Fluka, Milan, Italy >96%; Au/NaBH_4_ 1/4 mol/mol) solution was added to the solution under vigorous magnetic stirring. A ruby red Au(0) sol was immediately formed. An UV–visible spectrum of the Au sol was recorded to check the complete AuCl_4_^−^ reduction and the formation of the plasmon peak, of Au(0) nanoparticles.

#### 4.1.2. Pd nanoparticles Preparation.

Solid Na_2_PdCl_4_ (Aldrich, 99.99% purity; Pd 0.043 mol) and PVA solution (1% *w*/*w*) (Pd/PVA 1:1 *w*/*w*) were added to 100 mL of H_2_O. After 3 min, NaBH_4_ (Pd/NaBH_4_ = 1/8 mol/mol) solution was added to the yellow-brown solution under vigorous magnetic stirring. The brown Pd(0) sol was immediately formed. An UV–visible spectrum of the Pd sol was recorded to check the complete PdCl_4_^2−^ reduction.

### 4.2. Bimetallic AuPd Nanoparticles

Solid NaAuCl_4_•2H_2_O (0.0258 mol) and Na_2_PdCl_4_ (0.0172 mol) (Au/Pd ratio 6:4 mol/mol) and PVA solution (1% *w*/*w*) (Pd/PVA 1:1 *w*/*w*) were added to 100 mL of H_2_O. After 3 min, NaBH_4_ (Pd/NaBH_4_ = 1/8 mol/mol) solution was added to the yellow-brown solution under vigorous magnetic stirring. The brown AuPd(0) sol was immediately formed. An UV–visible spectrum of sols were recorded on a HP8453 spectrophotometer (Hewlett-Packard, Milan, Italy) in H_2_O between 200 and 800 nm, in a quartz cuvette to check the complete metal reduction.

### 4.3. Electrochemical Characterization

Electrochemical characterizations were carried out in a conventional three electrodes cell, by using a saturated calomel, a Pt wire and a modified glassy carbon (GC, 3 mm diameter, AMEL, Milan, Italy) as reference, counter and working electrodes, respectively. The modified glassy carbon was prepared depositing 20 µL of nanoparticles sol on the top of the electrode by a Kartell (Noviglio, Milan, Italy)automatic pipette. The GC electrode was previously polished with diamond synthetic monocrystalline nanopowder (Aldrich, 1 μm, 99.9%) on a DP-Nap wet cloth (Struers, Arese-Milan, Italy) and rinsed in water. Milli-Q water (18 MΩ cm^−1^, Millipore, Billerica, MA, USA) was used for the preparation of all the solutions.

Cyclovoltammetric investigations were performed using an Autolab PGSTAT 30 (EcoChemie, Utrecht, The Netherlands) equipped with NOVA (Advance Electrochemical Software, Utrecht, The Netherlands), in 0.1 M KCl or 0.1 M H_2_SO_4_ as supporting electrolytes, at a scan rate of 100 or 500 mV·s^−1^. Electrochemical Impedance Spectroscopy (EIS) measurements were carried out in 0.1 M KCl in the presence of the redox probe K_4_[Fe(CN)_6_] (3 mM, Sigma Aldrich Analytical Grade) using the PGSTAT 30 (Autolab, Utrecht, The Netherlands) equipped with a Frequency Response Analyzer (FRA). Range of frequencies was fixed between 0.1 Hz and 65,000 Hz with an amplitude of 10 mV. Impedance spectra were fitted using electrical equivalent circuits generated with ZView 3.2 software (Scribner Associates Inc., Southern Pines, CA, USA).

### 4.4. Catalytic Tests

Glycerol oxidation was carried out in a 30 mL glass reactor equipped with a thermostat and an electronically controlled magnetic stirrer connected to a 5000 mL reservoir charged with oxygen (3 atm.). The oxygen uptake was followed by a mass-flow controller connected to a PC through an A/D board, plotting a flow time diagram.

Glycerol 0.3 M, the sol (substrate/total metal = 1000 mol/mol) and 4 equivalent of NaOH were mixed in distilled water (total volume 10 mL). The reactor was pressurized at 3 atm of oxygen and set to 50 °C. Once this temperature was reached, the reaction was initiated by stirring. Samples were removed periodically and analyzed by high-performance liquid chromatography (HPLC) using a column (OA-10308, 300 mm–7.8 mm, Alltech, Milan, Italy) with UV and refractive index (RI) detection to analyze the mixture of the samples. Aqueous H_3_PO_4_ solution (0.1 wt %) was used as the eluent. Products were identified by comparison with the original samples.

## 5. Conclusions

CV and EIS characterisations of AuPd nanoparticles and the comparison with those of single metals established that there is a net electronic effect between the two metals. These characterization techniques can be easily used for establishing the nature of the bimetallic systems. Moreover, combining the catalytic results with the electrochemical characterization we concluded that the higher activity of the AuPd bimetallic system in the liquid phase oxidation of glycerol is due to a compromise between the stability of the oxidic species (decreased with respect to Pd) and the facility of hydride formation (increased with respect to Au). This balance creates the observed synergistic effect between Au and Pd.

## Figures and Tables

**Table 1:** Catalytic results in liquid phase oxidation of glycerol.

Catalyst ^a^	Mean Particle Size (nm)	Activity ^b^	Selectivity (%) ^c^					
			Glyceric Acid	Glycolic Acid	Tartronic Acid	Formic Acid	Lactic Acid	Oxalic Acid
Au_PVA_	2.6	1065	64	23	1	5	3	4
Pd_PVA_	2.8	299	85 ^d^	2 ^d^	4 ^d^	3 ^d^	4 ^d^	2 ^d^
AuPd_PVA_	3.4	4264	83	3	7	4	1	2

^a^ Glycerol 0.3 M in water; 4eq of NaOH; metal/alcohol = 1/1000 mol/mol; 300 kPa O_2_; T = 50 °C; ^b^ Calculated as mol of glycerol converted per hour per mol of metal, calculated after 15 min reaction; ^c^ selectivity at 80% of conversion; ^d^ selectivity at 20% of conversion.
